# Complications From Operator Modification of the Esophageal Temperature Probe

**DOI:** 10.7759/cureus.30388

**Published:** 2022-10-17

**Authors:** Peter L Kovacs, Zachary Deutch

**Affiliations:** 1 Anesthesiology, University of Florida, Jacksonville, USA

**Keywords:** patient harm from medical device, modified medical device, laryngeal mask airway anesthesia, general endotracheal anesthesia, modified esophageal temperature probe, esophageal temperature probe

## Abstract

Intraoperative temperature monitoring of surgical patients is an important aspect of perioperative care. Central core temperature monitoring is often accomplished using an 18 French esophageal device inserted through the mouth into the esophagus, while patients are undergoing general anesthesia. Placement of a modified esophageal temperature probe (i.e. with the protective plastic covering removed) into the nasopharynx of a patient may cause significant patient harm by injuring the nasal mucosa and/or turbinates. An internal survey of current practice at our academic institution reveals that 78% of anesthesia providers modify the esophageal temperature probes, leading to an 11% injury incidence. A practical solution to avoid complications is to place a pediatric size 9 French esophageal temperature probe into the nasopharynx to monitor the central core temperature.

## Introduction

General anesthesia impairs thermoregulatory control [1]. As thermoregulation is needed for the normal functioning of physiologic mechanisms, accurate measurement and management during anesthesia are important for optimizing perioperative outcomes [2]. In an adult patient undergoing general anesthesia, heat loss (radiation, convection, evaporation, and conduction) and heat redistribution are two important reasons for a decrease in core body temperature. When significant temperature changes may occur (eg, during long surgical procedures or procedures that include active cooling and rewarming such as cardiac surgery), it is recommended that core temperature be monitored [3]. Reliable locations to monitor core temperature even with rapid changes in body temperature include the pulmonary artery, tympanic membrane, nasopharynx, and distal esophagus. Skin temperature measurement is greatly impacted by peripheral perfusion and is usually lower than core temperature. However, the difference between skin and core temperature is not well correlated, especially when the temperature is changing rapidly. Rectal temperature lags with rapid temperature changes. Bladder temperature is impacted by urine flow and can be especially unreliable with low urine output.

Perioperative hypothermia is common and causes impaired pharmacodynamics, surgical site infections, blood loss and coagulopathy, transfusion requirements, thermal discomfort, prolonged recovery, and prolonged duration of hospitalization [4-6]. Perioperative hyperthermia may be associated with fever, malignant hyperthermia, sepsis, stroke, and toxicology [7-9].

Core temperature monitoring can be measured with an 18 French esophageal temperature probe placed through the mouth into the esophagus when performing general anesthesia with an endotracheal tube (ETT). Placement of an operator-modified (removal of protective covering) 18 French esophageal temperature probe into the nasopharynx of an anesthetized patient using a laryngeal mask airway (LMA) may cause patient harm by injuring the nasal mucosa and/or nasal turbinate. The use of an LMA can prevent the placement of the temperature probe into the esophagus due to the position of the laryngeal mask in the pharynx and occluding the esophagus.

An internal survey and quality review of our anesthesia department was conducted with 50 total respondents (physicians, nurse anesthetists, and anesthesiologist assistants). The responses revealed that operator modification of esophageal temperature probes is a common practice (78% of respondents) and it results in injury to the mucosa with bleeding 12% of the time.

## Case presentation

**Figure 1:**
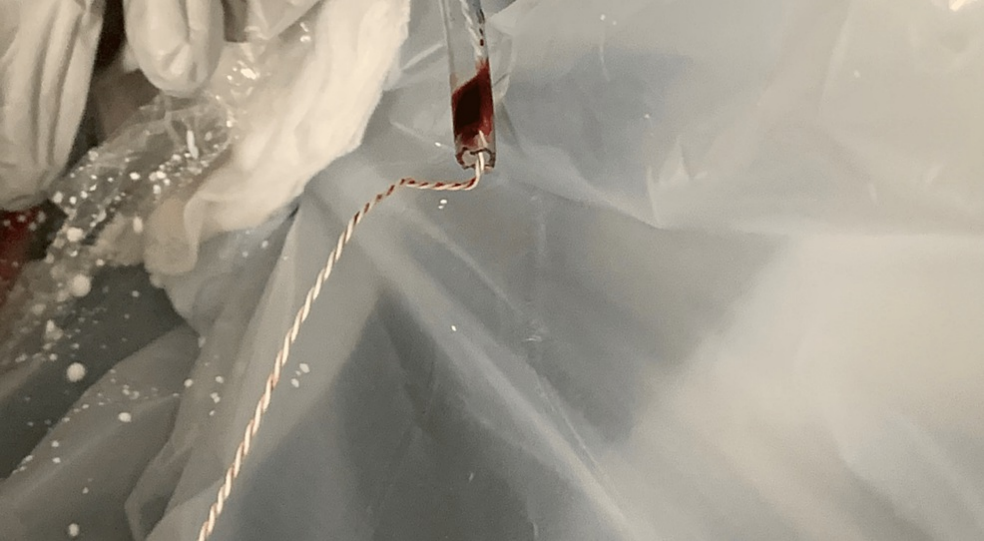
Modified 18 French esophageal temperature probe

A 35-year-old white male underwent an open reduction internal fixation of the right tibia. General anesthesia was provided with induction provided by fentanyl 100 mcg, lidocaine 50 mg, and propofol 200 mg. The patient's airway was secured with a size 5 LMA without incident. A modified 18 French esophageal temperature probe (Figure 1) was placed into the nasopharynx. Specifically, the plastic covering was removed in order to expose the monitoring wire and the temperature sensor itself. No trauma to the nasopharynx was noted upon insertion of the temperature probe. At the completion of the surgical procedure, the temperature probe was removed from the nasopharynx and significant blood (approximately 25-30 mL) was suctioned from the nasopharynx and the oropharynx. The pressure was applied to the nares with eventual slowing of blood flow. After several minutes, no more blood was noted in the nasopharynx or oropharynx. The patient was transported to the post-anesthesia care unit in stable condition with no other problems noted.

## Discussion

**Figure 2:**
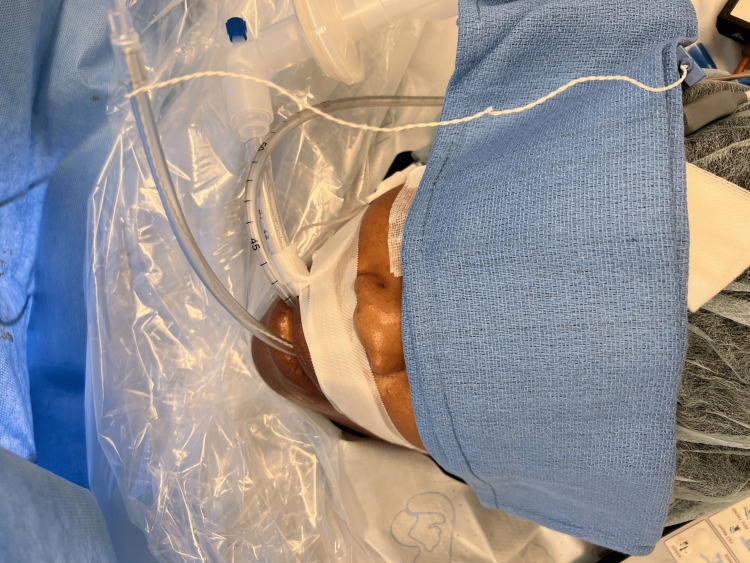
Endotracheal tube and oral 18 French esophageal temperature probe

When a patient undergoes a surgical procedure with general anesthesia, patient temperature monitoring is an important aspect of intraoperative care. This is often accomplished via an oral 18 French esophageal temperature that monitors central core temperature. If general anesthesia is used with an ETT, the 18 French esophageal temperature probe easily passes into the esophagus in a side-by-side fashion with the ETT (Figure 2). 

**Figure 3:**
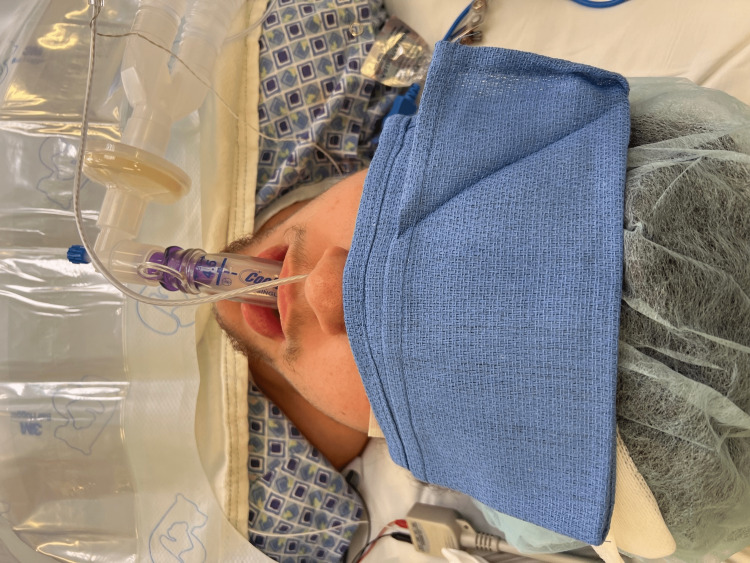
Laryngeal mask airway with 9 French esophageal temperature probe placed into nasopharynx

However, when an LMA device is used instead of an ETT, the esophageal temperature probe cannot be placed due to the size of the LMA and consequent physical occlusion of the esophagus. An internal survey of anesthesia providers at our institution revealed that it is not uncommon to physically alter the 18 French esophageal temperature probe and place this into the nasopharynx of the anesthetized patient. An acceptable alternative is to place the 9 French esophageal temperature probe into the nasopharynx (Figure 3).

As mentioned above, it is not uncommon to modify (remove the protective covering) the 18 French esophageal temperature probes to reduce their outer diameter and allow them to be more easily placed into the nasopharynx. This can be accomplished either by using scissors or by perforating the plastic sheath with a blunt needle and separating it from the wire/temperature sensor, leaving a serrated jagged plastic edge. In the case of our patient, the serrated edge of the operator-modified esophageal temperature probe lacerated the nasal mucosa. Previous case reports have documented injury with the use of esophageal temperature probes (uvular injury, bronchospasm, and esophageal laceration) [10-13], but no literature was found where an 18 French esophageal temperature probe was modified and then placed into an anesthetized patient.

A patient safety yellow belt project was initiated in our anesthesia department after review and evaluation by the quality improvement committee. Recommendations were made to the anesthesia providers during a quarterly quality improvement/patient safety conference. Modification and/or alteration of the 18 French esophageal temperature probe was discouraged. A solution presented was to use an unaltered pediatric size 9 French esophageal temperature probe inserted into the nasopharynx to monitor the central core temperature when an LMA is in place. Additionally, the availability of the 9 French esophageal temperature probes was to be ensured in all anesthetizing locations by anesthesia technicians during routine stocking of supplies.

## Conclusions

Monitoring the central core temperature of surgical patients under general anesthesia is an important aspect of good perioperative care. Operator modification of an 18 French esophageal temperature probe and placing it into an anesthetized patient can lead to injury. An appropriate alternative is to use an unaltered pediatric-sized 9 French esophageal temperature probe and place this into the nasopharynx to monitor the central core temperature when an LMA is used.
